# Pilot implementation of a home-care programme with chlamydia, gonorrhoea, hepatitis B, and syphilis self-sampling in HIV-positive men who have sex with men

**DOI:** 10.1186/s12879-020-05658-4

**Published:** 2020-12-04

**Authors:** J. Leenen, C. J. P. A. Hoebe, R. P. Ackens, D. Posthouwer, I. H. M. van Loo, P. F. G. Wolffs, N. H. T. M. Dukers-Muijrers*

**Affiliations:** 1 Department of Sexual Health, Infectious Diseases and Environmental Health, South Limburg Public Health Service (GGD Zuid Limburg), Heerlen, the Netherlands; 2 Department of Medical Microbiology, Care and Public Health Research Institute (CAPHRI), Maastricht University Medical Centre (MUMC+), Maastricht, the Netherlands; 3 Department of Social Medicine, Care and Public Health Research Institute (CAPHRI), Maastricht University Medical Centre (MUMC+), Maastricht, the Netherlands; 4 Department of Integrated Care, Maastricht University Medical Centre (MUMC+), Maastricht, the Netherlands; 5 Department of Internal Medicine, Maastricht University Medical Centre (MUMC+), Maastricht, the Netherlands; 6 Department of Health Promotion, Care and Public Health Research Institute (CAPHRI), Maastricht University Medical Centre (MUMC+), Maastricht, the Netherlands

**Keywords:** Home sampling, STI, MSM, HIV care, Implementation

## Abstract

**Background:**

Not all men who have sex with men (MSM) at risk for sexually transmitted infections (STIs) and human immunodeficiency virus (HIV) infection currently receive sexual healthcare. To increase the coverage of high-quality HIV/STI care for MSM, we developed a home-care programme, as extended STI clinic care. This programme included home sampling for testing, combined with treatment and sexual health counselling. Here, we pilot implemented the programme in a hospital setting (HIV-positive MSM) to determine the factors for the successful implementation of STI home sampling strategies.

**Methods:**

Healthcare providers from the HIV hospital treatment centre (Maastricht) were invited to offer free STI sampling kits (syphilis, hepatitis B, [extra]genital chlamydia and gonorrhoea laboratory testing) to their HIV-positive MSM patients (March to May 2018). To evaluate implementation of the program, quantitative and qualitative data were collected to assess adoption (HIV care providers offered sampling kits to MSM), participation (MSM accepted the sampling kits) and sampling-kit return, STI diagnoses, and implementation experiences.

**Results:**

Adoption was 85.3% (110/129), participation was 58.2% (64/110), and sampling-kit return was 43.8% (28/64). Of the tested MSM, 64.3% (18/28) did not recently (< 3 months) undergo a STI test; during the programme, 17.9% (5/28) were diagnosed with an STI. Of tested MSM, 64.3% (18/28) was vaccinated against hepatitis B. MSM reported that the sampling kits were easily and conveniently used. Care providers (hospital and STI clinic) considered the programme acceptable and feasible, with some logistical challenges. All (100%) self-taken chlamydia and gonorrhoea samples were adequate for testing, and 82.1% (23/28) of MSM provided sufficient self-taken blood samples for syphilis screening. However, full syphilis diagnostic work-up required for MSM with a history of syphilis (18/28) was not possible in 44.4% (8/18) of MSM because of insufficient blood sampled.

**Conclusion:**

The home sampling programme increased STI test uptake and was acceptable and feasible for MSM and their care providers. Return of sampling kits should be further improved. The home-care programme is a promising extension of regular STI care to deliver comprehensive STI care to the home setting for MSM. Yet, in an HIV-positive population, syphilis diagnosis may be challenging when using self-taken blood samples.

**Supplementary Information:**

The online version contains supplementary material available at 10.1186/s12879-020-05658-4.

## Background

Men who have sex with men (MSM) are at increased risk of acquiring human immunodeficiency virus (HIV) infections and sexually transmitted infections (STIs) [1]. STI continues to be a growing epidemic among MSM [2], particularly for those living with HIV. Integration of STI testing and control strategies with HIV testing and care is imperative to stop STI transmission at the population level and to enable optimal HIV/STI patient management [3].

Dutch national guidelines recommend the routine, i.e. up to four times a year [4] testing of HIV-positive MSM for syphilis, genital, anorectal, and oropharyngeal *Neisseria gonorrhoea* (NG) infections, for genital *Chlamydia trachomatis* (CT) infection, and, after self-reporting the symptoms of extragenital infection, receptive anal sex, or oral sex, for extragenital chlamydial infection [5]. However, not all MSM receive appropriate sexual healthcare services, despite testing guidelines and existing high-quality sexual healthcare [4, 6, 7]. For HIV positive MSM, STI test practice in HIV care is not always fully implemented [3] and is furthermore likely to miss extragenital chlamydia cases as these are frequently asymptomatic and frequently observed in the absence of reported anal sex [8]. For example, in a US HIV care hospital setting, STI screening in the hospital setting was only 2.0–8.5% [9].

In the Netherlands, STI clinics provide comprehensive sexual healthcare for MSM, which includes free-of-charge testing for HIV, hepatitis B (HBV), syphilis, and (extra)genital bacterial STI, STI treatment, HIV care referral to the hospital HIV treatment centre, partner notification, and sexual health counselling. HIV-positive people are treated at HIV treatment clinics. Here, care providers can also offer STI tests to their patients. However, there are no specific HIV (hospital) clinic guidelines that recommend routine STI screening for HIV positive MSM patients during regular HIV care visits; patients are tested only when they are considered at risk for STI’s. MSM can also get tested at the general practitioner (GP) for STIs. Depending on the type of health insurance, MSM may have to pay for the visit and tests and GPs testing guidelines only recommend extragenital testing based on sexual history and reported symptoms.

Suboptimal STI testing of MSM in the HIV care setting has several barriers at the care provider level and patient level. For HIV care professionals, the following barriers are encountered when performing STI testing to MSM patients: insufficient funds for STI screening, competing priorities (insufficient time for STI testing), and professionals’ uncomfortable feeling when discussing patients’ sexual practices [10, 11]. HIV-positive MSM may seek STI care outside the HIV clinic, including their general practitioner or an STI clinic, because of the following reasons: STI testing in an STI clinic is easier accessible compared to an HIV clinic, wanting to maintain anonymity, and more frequent testing can be performed in an STI clinic than in an HIV clinic [10].

To reach out to a significant number of MSM (HIV-positive and HIV-untested and negative MSM) with comprehensive HIV/STI care, we recently developed a regional home-care programme, as an extension of regular STI clinic care. The programme encourages MSM to undergo HIV/STI testing and be treated, using home sampling for comprehensive testing on HIV (restricted to HIV-negative or untested MSM), HBV (restricted to unvaccinated MSM), syphilis, and anorectal, urogenital, and oropharyngeal CT and NG. The programme was systematically developed, in close collaboration with its users, according to the intervention mapping strategy, reported elsewhere (future reference), to address and overcome barriers to HIV/STI testing. Self-sampling at home (i.e. home sampling) is the central component of our programme as it has been proven to be an effective additional strategy to increase STI testing uptake [12, 13]. Self-sampling at home makes testing convenient, increasing patient autonomy, saving time for care providers, and decreasing barriers for MSM in undergoing regular testing and for providers in offering STI testing to their MSM patients. Professionals in HIV treatment clinics perceived home sampling tests as time-saving for providers, overcoming patient discomfort and enabling increased patient access to testing [14].

In our home-care programme, home sampling is combined with eHealth technologies, which means that semi-automatic and semi-tailored text messages methods are used to improve response and enable better patient management. A large body of evidence has emerged displaying the effectiveness of text messaging in HIV/STI control [15]. The programme offers high-quality regular STI clinic care, and testing is linked to STI treatment, HIV care referral, partner notification, and sexual health counselling. Our home-care programme is designed for implementation as extension to regular care in various sexual healthcare settings, including STI clinics, but also including general practices (GP), and hospital HIV treatment centres.

In this paper, we describe the pilot implementation of this newly developed home-care programme within the hospital setting of the HIV treatment centre. This study aimed to evaluate this pilot implementation regarding its test usage and logistics and to reveal the experiences of the users (HIV-positive MSM) and implementers (hospital HIV treatment providers and STI clinic professionals). The findings will further aid in the optimisation of the programme and can provide further insights to sexual healthcare providers who intend to use home sampling strategies to improve the testing uptake in MSM.

## Methods

### Components of the home-care programme and implementation

Home sampling kits for CT, NG, syphilis, and HBV were offered by healthcare providers from the hospital HIV treatment clinic in Maastricht to their MSM patients when they routinely attended HIV care (March 2018 to May 2018), regardless of their STI testing history. Healthcare providers could offer a sampling kit to their HIV positive patients when they were 18 years or older, understood Dutch or English language, and ever had sex with men. When a patient accepted a sampling kit, his telephone number was documented because a text message reminder will eventually be sent to the patient once the sampling kit was not received by the laboratory within 2 weeks. When needed, a second reminder will be sent 2 weeks thereafter. After self-taking the samples and completing the accompanying online questionnaire, participants could return the samples to the laboratory for testing.

After the participants returned the self-taken materials and questionnaires, further patient management was handled by the STI clinic. The STI clinic communicated the laboratory test results to the participants via routine STI clinic protocol. This entailed a text message in cases of a negative result and phone call in case of a positive result or when further contact was required. Participants were invited to attend the STI clinic when needed, such as for treatment, partner notification, counselling, and further diagnostics, when the self-taken sample was deemed inadequate. The role of the STI clinic was to oversee the implementation process and to manage all logistics and patient STI care.

### Data collected for evaluation purposes

During the pilot implementation, HIV treatment providers provided coded and aggregated data on age and country of birth (aggregated for MSM who accepted a test kit and those who did not) and a frequency list of provider’s reasons for not offering a sample kit and MSM’s reasons for refusing an offered sample kit. Country of birth was categorized in western (born in Europe, Northern America, Oceania, Japan or Indonesia, according to the definition of Statistics Netherlands (https://www.cbs.nl/en-gb)) and non-western countries.

When MSM refused a sample kit, the healthcare provider asked for the reason (open-question). For feasibility and time reasons for the healthcare provider, the healthcare provider filled in the patients response on a pre-specified list, with also an open-text response, if none of the pre-specified options were suitable. Due to privacy issues only aggregated data was available on reasons for declining for this study.

MSM who underwent HIV/STI testing provided quantitative data on their socio-demographics, STI testing history, risk behaviour, and experiences with the home sampling kit by completing the online questionnaire. The questionnaire was available in Dutch and English. The content of the online questionnaire was similar to the medical history form regularly obtained at STI clinic care, with the addition on questions on user experience of the home sampling kit (See Additional file 1 for a list of questions asked).

Further data collected included quantitative process data on test-kit use and return and STI diagnostic data.

We also collected qualitative information (from our regular group meetings) regarding the users’ experiences in the logistics (acceptability and feasibility) of the implementation process from all professionals involved. These included the healthcare providers of the HIV hospital clinic (offering STI kits), logistical team members (handling the sampling kits), laboratory staff (testing the samples), and care providers (nurses, doctors, assistants) of the STI clinic (providing patient care).

### Sampling-kit content and laboratory testing

Each sampling kit contained an information package, with information about HIV/STI in general, and instructions on home sampling procedures and on how to return the samples. Kits included a swab for oral CT and NG, a swab for anorectal CT and NG, a urine collection tube for genital CT and NG and for syphilis and HBV testing, and a small blood collection tube with two finger prick sticks for capillary blood sampling. Sampling kits could be returned free of charge to the STI clinic of South Limburg via regular postal mail. Samples were tested in the medical microbiology laboratory at Maastricht University Medical Centre. Swabs and urine were processed with a polymerase chain reaction for CT and NG (Roche Cobas 4800, Roche Diagnostics, Basel, Switzerland). A syphilis screening test (*Elecsys® syphilis immunoassay*, Roche, Basel, Switzerland) was performed. However, when MSM reported a history of syphilis in the standardised questionnaire, a rapid plasma reagin reditest (Biokit, Barcelona, Spain) was performed to measure the activity of the infection by antibody titre [16].

When MSM stated in the standardised questionnaire that they were HBV unvaccinated, HBV serology was performed on the blood sample. In case of a positive anti-hepatitis B core antigen test, hepatitis B surface antigen (HBsAg) test and anti-HBs (HBsAg II and anti-HBs II, Roche, Basel, Switzerland) were performed to determine HBV status.

### Implementation evaluation

The quantitative evaluation included descriptive statistics to assess the proportions of [1] adoption by providers (i.e. HIV care providers offering sampling kits to their MSM patients and reasons for not offering a sampling kit, [2] participation (i.e. the sampling-kit acceptance by MSM and reasons for not accepting a sampling kit), and [3] other indicators such as the proportion of test kits returned, STI diagnosis, test history, and HBV vaccination. We reported the user experiences of MSM who underwent testing.

Furthermore, regarding the qualitative evaluation of user experiences, we described the barriers in the implementation process during the evaluation meetings with key professional stakeholders.

## Results

### Adoption (offering tests by care providers)

**Fig. 1:**
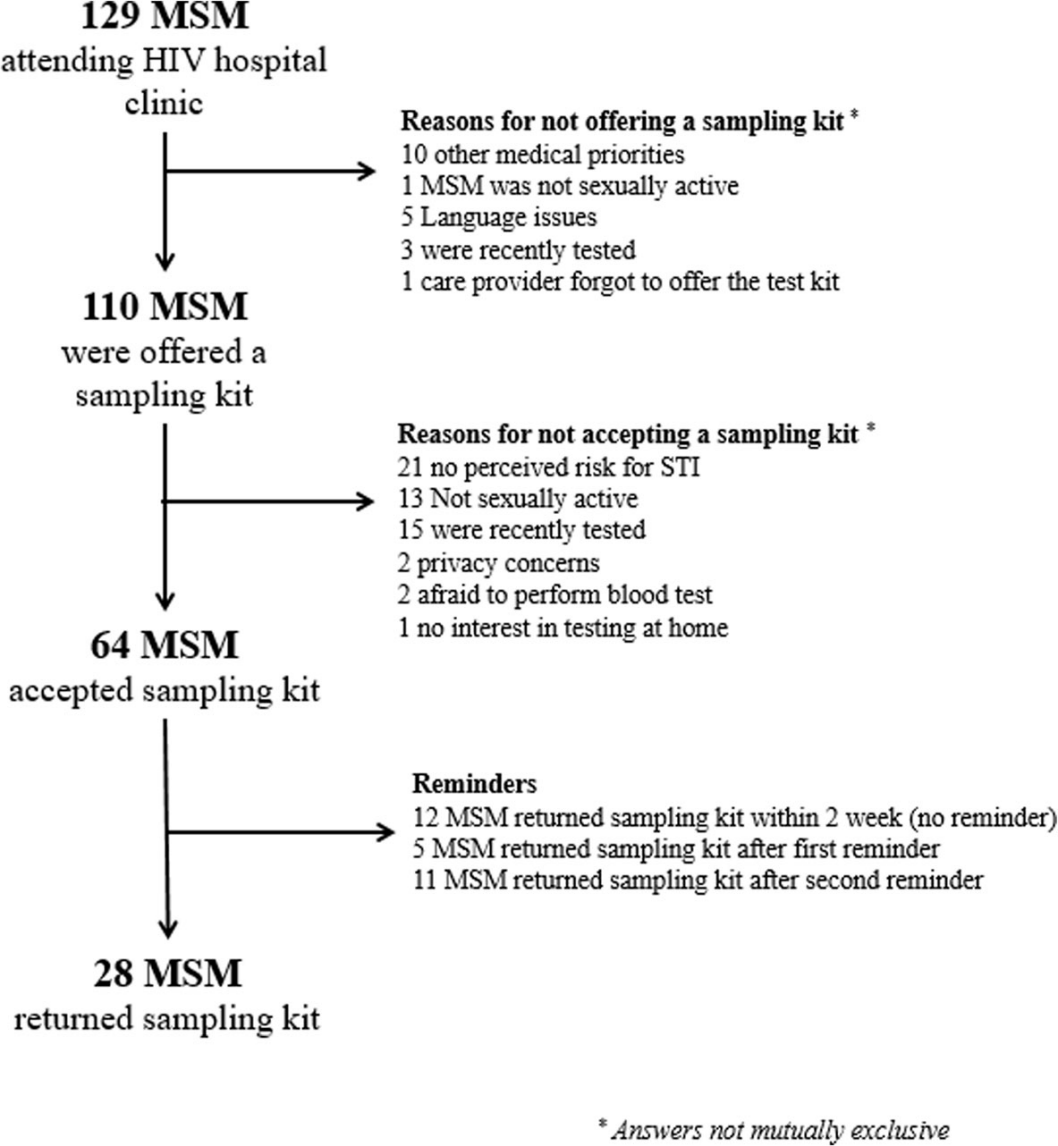
Flowchart of adoption, participation, and return of home sampling kits in a hospital setting (Human Immunodefiency Virus treatment centre)

Of the 129 MSM who attended the HIV treatment clinic for HIV care (see Fig. 1), mean age was 46 years and 60.6% MSM had a western country of birth. 110/129 MSM (85.3%) were offered a home sampling kit. Reasons for not offering a sampling kit by providers were as follows: other medical priorities had to be considered (10/19, 52.6%), MSM did not understand the test instructions’ language (Dutch or English) (5/19, 26.3%), MSM were recently tested for STI (3/19, 15.8%), MSM were not sexually active (1/19, 5.3%), or the care provider forgot to offer the sampling kit (1/19, 5.3%). The mean age of MSM who were offered a home sampling kit was 47 years, and 84.5% of MSM had a western country of birth (93/110) (Table 1).

**Table 1:** Demographic data on adoption, participation and return (aggregate level)

	Offered sampling kit			Accepted sampling kit			Returned sampling kit		
	n	mean age	Western country of birth (%)	n	mean age	Western country of birth (%)	n	Mean age	Western country of birth (%)
**Yes**	110	47	84.5	64	46	87.5	29^a^	50^b^	89.3^a^
**No**	19	43	57.9	46	47	78.3	35	na	na

^a^One patient refused contact after returning sampling kit and was excluded in further analyses

^b^*n* = 25, 1 MSM excluded, missing data in 3 MSM

*na* not available; Demographic data from MSM who accepted a sampling kit, but did not return the sampling kit was not available due to medical ethical considerations

### Participation (accepting tests by men who have sex with men [MSM])

Of the 110 MSM who were offered a sampling kit, 64 (58.2%) accepted a sampling kit (Fig. 1). Reasons for not accepting a sampling kit were as follows: no perceived risk for STI (21/46, 45.7%), recently tested for STI at the STI or GP’s clinic (15/46, 32.6%), or were not sexually active (13/46, 28.3%). The mean age of MSM who accepted a home sampling kit was 46 years, and 87.5% of the MSM had a western country of birth (56/64) (Table 1).

### Return (sampling-kit return by MSM who accepted the home sampling kit)

A total of 29 out of the 64 (45.3%) MSM used and returned the sampling kit; one participant refused contact after returning the sampling kit and was subsequently excluded in further analyses. Moreover, 12 out of the 28 (42.9%) MSM returned their sampling kit within 2 weeks, without a text message reminder. The remaining MSM (*n* = 51) received a first text message reminder 2 weeks after receiving the sampling kit. Furthermore, 5 out of the 51 MSM returned their sampling kit within 2 weeks after the first reminder, and the remaining MSM (*n* = 46) received a second text message 2 weeks after the first reminder. Additionally, 11 out of the 46 MSM returned their sampling kit within 6 weeks. In total, 16 out of the 28 (57.1%) MSM returned their sampling kit after receiving a text reminder. The mean age of MSM who returned the sampling kits was 50 years (*n* = 25, missing data in 3 MSM) and 89.3% had a western country of birth (Table 1).

### Test history, sample adequacy, and sexually transmitted infection (STI) diagnosis in testers

Of the 28 MSM who used a sampling kit, 10.7% (*n* = 3) were never tested before for STI (other than HIV) and 10 (35.7%) tested recently (in the past 3 months) (Table 2). Moreover, 64.3% (*n* = 18) of MSM were HBV vaccinated.

**Table 2:** Characteristics of the testers and their experiences with home sampling

	n (%)
Self-reported test history (*N* = 28)	
Never tested for STI (other than HIV)	3 (10.7)
Tested for STI in the past 3 months	10 (35.7)
Tested for STI in the past 3–12 months	9 (32.1)
HBV vaccinated	18 (64.3)
STI diagnosed (*N =* 28)	
Newly diagnosed STI (in home-care programme usinghome sampling)	5 (17.9)
Successful sampling and testing (*N =* 28)	
Successful oral CT and NG testing	28 (100)
Successful genital CT and NG testing	28 (100)
Successful anorectal CT and NG testing	28 (100)
Blood sample > 100 μl	23 (82.1)
Successful syphilis diagnosis (regular care)	17 (60.7)
Successful syphilis diagnosis (individual approach required)	5 (17.9)
Experience with home sampling (*N* = 23)^a^	
Test instructions: (very) clear	22 (95.7)
Home sampling would be their test method of choicein the future	14 (60.9)
Would give a home sampling test to a peer (friend orsex partner)	17 (73.9)
Benefits of home sampling: testing when convenientand at own time	18 (64.3)
Benefits of home sampling: testing at home	13 (46.4)
Benefits of home sampling: no transportation required	13 (46.4)

^a^Missing questionnaire data from five individuals

All (100%) self-taken urine and swab materials were sufficient for further laboratory processing and testing. A total of 23 (82.1%) self-taken blood samples contained more than 100 μl of blood and thus were sufficient for HBV and syphilis screening, and these samples also had sufficient residual blood for HIV screening. However, 18 out of the 28 (64.3%) MSM reported a history of syphilis, requiring more sample materials for syphilis diagnostic work-up; 8 out of the 18 (44.4%) MSM had insufficient material and were further managed with tailored care (e.g. further tested at the STI clinic). All were negative for a new syphilis infection.

Using home-sampling in our programme, 5 out of the 28 (17.9%) MSM were newly diagnosed with one or more STI (i.e. genital CT, rectal CT, oral NG, rectal NG). All diagnosed MSM were asymptomatic treated and counselled at the STI clinic.

### User experiences: MSM who underwent testing

The majority of MSM reported that the instructions provided in the sampling kit were clear. The main benefit of home sampling was the convenience in taking the samples (see Table 2). One MSM reported unclear blood sampling instructions. A number of MSM reported that home sampling (rather than sampling at clinic) would be their test method of choice in the future; MSM would not only recommend such sampling to a peer but also would provide a home-sampling kit to other peers themselves. Some MSM reported, to their HIV healthcare provider, that the online questionnaire was significantly extensive, and had concerns about their privacy.

### User experiences: hospital providers

HIV care providers reported that overall, offering sampling kits was an easy and quick way to offer an STI test to a patient. However, offering sampling kits sometimes led to additional questions from patients during their regular HIV treatment centre visit (normal duration, 20 min), which was considered time consuming as it could take up HIV care providers’ (nurses and physicians) additional 5 min’ extra time. As an addition to a future programme, providers stated they would prefer the possibility of handing out the sampling kits to their patients’ partners, by providing their patients with an extra sampling kit, as this was specifically requested by a few patients.

### User experiences: STI clinic providers

STI clinic care providers reported that overall, home sampling kits could be a valuable addition to regular STI care for MSM related to costs and time; however, some components needed to be improved. STI clinic providers handled the logistics of the programme and STI patient care. Nurses handling the sampling kits felt it was time consuming when the sampling materials were insufficient or when the standardised questionnaire was incomplete as this required additional effort from the clinic nurse (e.g. when syphilis or HBV vaccination status was unknown). Physicians from the STI clinic acknowledged that syphilis diagnosis in MSM who had a history of syphilis can be complicated. First, a number of testers did not provide sufficient blood samples for a full diagnostic work-up; hence, a nurse communicated with the testers for an additional STI clinic visit. Second, even in the case of sufficient self-taken blood samples, the interpretation of the syphilis laboratory tests is difficult when no preceding syphilis test results are available for this patient. Thus, the STI clinic providers (after MSM consent) had to perform further actions such as searching the MSM’s medical records, initiating phone calls to GP/HIV treatment specialist, and performing an additional HIV/STI testing at the STI clinic. Hence, nurses suggested that obligatory questions should only be included as part of the data collection methodology so that missing necessary data will be avoided. Suggestions regarding effective patient management in case of a syphilis history were not reported because this has been also been encountered in routine face-to-face clinical practice.

## Discussion

In this study, we performed a pilot implementation of a home-care programme to improve the HIV/STI care of MSM using home sampling kits combined with high-quality sexual healthcare. In addition to previous studies, who assess and acknowledge the use of home sampling for bacterial STIs or HIV [12, 13, 17, 18], our home-care program includes bacterial STI, as well as HIV and syphilis testing, follow-up treatment and comprehensive sexual healthcare and can be sampled at home and send with postal mail for laboratory testing.

Here, the programme was pilot-implemented in the hospital HIV treatment setting to improve the uptake of STI testing and sexual healthcare in HIV-infected MSM. Our evaluation revealed that adoption of the programme by HIV care providers was adequate, that is, 85.3% of patients were offered a home sampling kit. Participation, that is, acceptance of sampling kits by MSM, was 58.2%, and sampling kit return was 43.8%. Samples that were self-collected were generally adequate, but establishing a syphilis diagnosis was complex in case a patient reported a history of syphilis. Several barriers at the logistic and the care provider level were reported, suggesting that further optimisation of our home-care programme for MSM with comprehensive sexual healthcare is required.

In developing the programme and during its implementation, regular meetings and in-person contact were established between the care providers (implementers) and the programme developers, which is considered essential to sustain and promote the use of the programme. We involved key stakeholders and implementers already in the early development phase of the programme to tailor the needs of care providers, share knowledge, create trust, and work on a shared goal for the project [19]. With these steps, we enhanced the implementation behaviour [future reference].

Our implementation pilot aimed to test the logistics of the programme components, to assess acceptance and feasibility and user experiences, and to determine the barriers of the programme.

The programme was established using home sampling methods, which are considered important in increasing the test uptake. Our pilot implementation confirmed that the use of text message reminders was important to increase the sampling-kit return [20, 21].

MSM involved in the programme reported a positive attitude towards home sampling. Previous studies have also shown that self-sampling increases CT and NG testing in patients undergoing HIV/STI testing in HIV clinics [22]. Besides urine samples and anorectal and oropharyngeal swabs, the test kit included a blood sample to test for syphilis and HBV. Our study confirmed that most MSM considered finger prick blood sampling feasible and acceptable based on the previous studies [23, 24].

Home sampling kit collecting blood (allowing for syphilis, HBV, and HIV screening) samples is a unique addition to home sampling kits for chlamydia and gonorrhoea. Nevertheless, in HIV-positive patients, establishing the diagnosis of syphilis was difficult, and a suboptimal diagnosis can only be established when a single self-collected blood sample is used.

The proportion of MSM with previous syphilis infection was high (18/28, 64.3%) [25], and in these patients, a syphilis screening test (requiring a small amount of blood sample) is not required. However, self-taken blood sample was insufficient for a full syphilis work-up and diagnosis in 44.4% of the patients who had a history of syphilis (8/18). Hence, additional efforts (e.g. initiating phone calls to the involved patients, obtaining patients’ consent when searching their medical history, or additional blood drawing at the STI clinic) are required. Discussing these issues with the project team, the addition of a second self-taken blood tube in the sampling kit to obtain sufficient blood samples was not considered patient-friendly and hence not a desired solution. In a previous study, dried blood spot was used for syphilis screening [23]. However, this method was also not optimal as not all samples were adequately obtained. Hence, additional efforts (searching the patients’ medical history) are still required. Although development and implementation for syphilis home sampling is promising [23, 26, 27], it is also challenging. A large study from the UK with home sampling using capillary blood sampling found that only 54% of the samples contained sufficient blood for syphilis testing [28]. Although in our study, we had more samples (82.1%) that contained sufficient blood for testing, lack of knowledge on patient syphilis history made syphilis diagnosis difficult in this particular approach taken.

In MSM with a lower proportion of past syphilis, such as HIV-negative or unaware patients, a syphilis screening test is usually considered the test of choice. An additional non-blood (saliva) HIV screening tests to the test kit may be considered but may not be required as in our study 82.1% (23/28) would have had sufficient blood samples for both syphilis and HIV screening.

Despite the difficulty in diagnosing syphilis, the programme can be a valuable extension to public health and regular care to reach MSM who do otherwise not receive comprehensive and regular sexual healthcare.

Because of the provider’s and the MSM’s significant effort, a comprehensive STI diagnosis was achieved (including syphilis) for all patients. However, the following question remains: Will the complexity of syphilis diagnosis negatively affect the home-care programme’s effectiveness? Based on our evaluation meetings, when discussing these issues, the benefits of home-care programme for public health (reaching more untested MSM) and individual patient management (providing a valid test result immediately) created a significant tension between stakeholders. Hence, properly weighting the benefits of the home-care programme for public health and individual patient care is important. Cost-effectiveness studies may shed further light on this issue. Adding STI screening to regular care at HIV treatment centres can be cost-effective in the Netherlands [29].

We encouraged MSM to return their sampling kit by message reminders, which increased the return rate from 18.8% (12/64) to 43.8% (28/64). Other studies showed higher HIV/STI home sampling return rates (55–84.5%) [30–32]. In our study, more than half of the distributed sample kits were lost. The sampling-kit return rate could possibly be increased if MSM were initially required to perform several actions in order for them to receive the kit, for example, by initially committing themselves to complete the forms online, read the information about home sampling, and exert some effort in completing their online medical history before receiving a sample kit [30]. Another way to increase the sampling-kit return rate could be by using other distribution methods, for example, peer dissemination [33]. The effect of different distribution methods among MSM on sampling-kit return rate should be further explored.

This study has some limitations. First is the generalisability of results. This pilot study was conducted in HIV-positive MSM who were already enrolled in HIV care. Use and acceptability of the sampling kits could be different among the general MSM population, such as the use of syphilis testing considering that HIV-positive MSM with the highest proportion of previous syphilis underwent HIV/STI testing in this study. Second is the limited number of MSM included in this pilot implementation study. Considering the objectives of our study, the number of MSM who participated in the study was insufficient for further data analysis. Nevertheless, information from 25 MSM was valuable to give an insight in user experiences to home sampling. Implementing this programme in a larger group (e.g. HIV-negative MSM or MSM who are not enrolled in care) would provide more insight on the generalisability of results to the broader MSM population. Third is related to medical ethical considerations considering that the demographic information of MSM who did not participate in the study is not available. This information would give better insight in characteristics of those who did not accept or did not return a sampling kit and could be used to inform future work. More research is needed to assess reasons for not returning sampling kits to improve return rate in future home-sampling sexual healthcare. Our study group will assess if applying for a sampling kit online and subsequent sending reminders after receiving a sampling kit will increase return rate in a new implementation of the programme ‘Limburg4zero’, to reach the broader population of MSM.

## Conclusion

The home sampling programme increased STI test uptake and was considered acceptable and feasible for most MSM and their care providers and could be a valuable extension to current sexual healthcare. In an HIV-positive population, syphilis diagnosis may be challenging when only single self-taken blood sample is used. From a public health view, the home-care programme is promising to deliver comprehensive STI care in the home setting for MSM. Results from this pilot study could be used to optimise and implement home sampling for HIV/STI tests in the future.

## Supplementary Information

**Additional file 1: Appendix 1**. Online questionnaire for MSM using home sampling kit (English version).

## Data Availability

Due to the Dutch law of protection of personal information (wet bescherming personengegevens Wbp or personal Data Protection Act: http://wetten.overheid.nl/BWBR0011468/geldigheidsdatum_13-07-2015), it is not allowed to distribute or share any personal data that can be traced back (direct or indirect) to an individual. The data used in our study are third-party data, which cannot be traced back to an individual. The data used in this study are not publicly available. For permission, interested researchers are required to provide their name and institution to avoid misuse of this sensitive data and to align with the Dutch law of protection of personal information. Therefore, interested researchers may contact the head of the data-archiving (Helen Sijstermans: Helen.sijstermans@ggdzl.nl) to receive the data.

## Abbreviations

CT: *Chlamydia trachomatis*
GP: General practitioner
HIV: Human Immunodeficiency Virus
HBV: Hepatitis B virus
MSM: Men who have sex with men
NA: Not applicable
NG: *Neisseria gonorrhoeae*
PO: Performance objective
STIs: Sexually transmitted infections
