# Supplementary material for: Pilot implementation of a home-care programme with chlamydia, gonorrhoea, hepatitis B, and syphilis self-sampling in HIV-positive men who have sex with men
Source: BMC Infect Dis. 2020 Dec 4;20:925. doi: 10.1186/s12879-020-05658-4 (PMC7716461; doi:10.1186/s12879-020-05658-4)
Supplement: Supplementary file 1 — Additional file 1: Appendix 1. Online questionnaire for MSM using home sampling kit (English version). [file 12879_2020_5658_MOESM1_ESM.docx]

**Appendix 1. Online questionnaire for MSM using home sampling kit (English version).**

1. Your First name

____________________

1. Surname

____________________

1. Date of birth(dd-mm-yyyy)

____________________

1. In which country are you born

- *The Netherlands*
- *Belgium*
- *Germany*
- *France*
- *United kingdom*
- *other*

1. You selected other. In which country are you born?(Only if question 4 is answered by other)

____________________

1. In which country is your father born?

- *The Netherlands*
- *Belgium*
- *Germany*
- *France*
- *United kingdom*
- *other*

1. You selected other. In which country is your father born? (Only if question 6 is answered by other)

____________________

1. In which country is your mother born?
   - *The Netherlands*
   - *Belgium*
   - *Germany*
   - *France*
   - *United kingdom*
   - *other*
2. You selected other. In which country is your mother born? (Only if question 8 is answered by other)

____________________

1. The GGD would like to send you an SMS if all your test results are good. Do you agree?

- *Yes*
- *No*

1. The following date will be used to inform you about your test results or to send you a reminder. If you do not wish to receive an SMS, you will receive your test results by e-mail.
2. What is your phone number? (06-)

____________________

1. What is your e-mail address?

____________________

1. Your address (street name and number)

____________________

1. ZIP code

____________________

1. Place of residence

____________________

1. Are you currently studying?

- *No*
- *Yes*

1. What is your highest (completed) level of education?

- *No education*
- *Primary school Vocational*
- *Training level 1-2 Vocational*
- *Training level 3-4*
- *Higher secondary school*
- *University*

1. Have you ever been tested for a sexually transmitted disease? (multiple answers possible)

- *No*
- *Yes, for HIV*
- *Yes, for STD*

1. What was the result of your last HIV test? (only if question 19 answered by “Yes, for HIV”)

- *Negative*
- *Positive*

1. What was the date of this HIV test?(this can be an estimation)(only if question 19 answered by “Yes, for HIV”)

____________________

1. Where did you take an STD of HIV test and how long ago was this? (only if question 19 not answered by “no”)

|  | *In the past 3 months* | *In the past 6 months* | *In the last year* | *Longer than a year ago* | *I never tested here* |
| --- | --- | --- | --- | --- | --- |
| *At the GGD* |  |  |  |  |  |
| *At the GP (general practitioner)* |  |  |  |  |  |
| *At the hospital (specialist)* |  |  |  |  |  |
| *Test ordered on the internet / drug store* |  |  |  |  |  |

1. Did you ever have one of the following STD?

|  | *In the past year* | *Longer than 1 year ago* | *I never had this STD* |
| --- | --- | --- | --- |
| *Chlamydia* |  |  |  |
| *Gonorrhea* |  |  |  |
| *Herpes on the genitals* |  |  |  |
| *Warts on the genitals* |  |  |  |
| *Syphillis* |  |  |  |
| *Hepatitis B* |  |  |  |

1. You indicated to have had chlamydia. Where on your body was this? (multiple answers possible)(only if question 23.1 not answered by “I never had this STD”)

- *Oral (mouth/throat)*
- *Genital (private parts)*
- *Anal (anus)*
- *I don’t know*

1. You indicated to have had gonorrhea. Where on your body was this? (multiple answers possible)(only if question 23.2 not answered by “I never had this STD”)

- *Oral (mouth/throat)*
- *Genital (private parts)*
- *Anal (anus)*
- *I don’t know*

1. Did you had sex with men, women of both?
   - *Only with men*
   - *Only with women*
   - *Both*
2. Did you have sex with an HIV positive man in the last 6 months? (only if question 26 not answered by “only with women”)

- *No*
- *Yes*
- *I don’t know*

1. Did you have sex with several men together or shortly after each other (group sex)?(only if question 26 not answered by “only with women”)

- *No*
- *Yes*

1. With how many people did you have sex in the past 6 months?(enter a number)

*Men: ____________________*

*Women: ____________________*

1. Did you had in the past 6 months, during a relationship, also other sex partners?

- *No*
- *Yes*
- *Had no relation*

1. Did someone you were in a relationship with in the past months had one or multiple other sex partners during this relationship? (only if question 30 not answered “had no relation”)

- *No, that is very unlikely*
- *Possibly*
- *Yes, very certain*

1. Did you have vaginal sex in the past 6 months? (only if question 26 not answered by “only with men”)

- *No (proceed to question 34)*
- *Yes, always with a condom (proceed to question 34*
- *Yes, also without a condom with 1 partner*
- *Yes, also without a condom with more partners*

1. When was the last time you had vaginal sex without a condom? (only if question 26 not answered by “only with men”)

- *In the past 2 weeks*
- *In the past 3 months*
- *More than 3 months ago*

1. Did you RECEIVE (bottom) anal (but) sex in the past 6 months? (only if question 26 not answered by “only with women”

- *No (proceed to question 36)*
- *Yes, always with a condom (proceed to question 36)*
- *Yes, also without a condom with 1 partner*
- *Yes, also without a condom with multiple partners*

1. When was the last time you RECEIVED anal sex without a condom? (this can be an estimation) (only is question 26 not answered by “only with women”)

- *In the past 2 weeks*
- *In the past 3 months*
- *Longer than 3 months ago*

1. Did you GAVE (top) anal (but) sex to men in the past 6 months? (only if question 26 not answered by “only with women”)

- *No (proceed to question 38)*
- *Yes, always with a condom (proceed to question 38)*
- *Yes, also without a condom with 1 partner*
- *Yes, also without a condom with multiple partners*

1. When was the last time you GAVE anal sex to men without a condom? (this can be an estimation)(only if question 26 not answered by “only with women”)

- *In the past 2 weeks*
- *In the past 3 months*
- *Longer than 3 months ago*

1. Did you gave anal (but) sex to women in the past 6 months? (only is question 26 not answered by “only with men”)

- *No (proceed to question 40)*
- *Yes, always with a condom (proceed to question 40)*
- *Yes, also without a condom with 1 partner*
- *Yes, also without a condom with multiple partners*

1. When was the last time you GAVE anal sex to women without a condom?(this can be an estimation) (only if question 26 not answered by “only with men”)

- *In the past 2 weeks*
- *In the past 3 months*
- *Longer than 3 months ago*

1. Did you have oral (mouth) sex in the past 6 months?

- *No (proceed to question 42)*
- *Yes, always with a condom (proceed to question 42)*
- *Yes, also without a condom with 1 partner*
- *Yes, also without a condom with multiple partners*

1. When was the last time you had oral sex without a condom?(this can be an estimation)

- *In the past 2 weeks*
- *In the past 3 months*
- *Longer than 3 months ago*

1. Have you ever received money or other goods for sex?

- *No (proceed to question 44)*
- *Yes, longer than 6 months ago (proceed to question 44)*
- *Yes, in the past 6 months*

1. What did you receive in exchange for sex in the last 6 months?

- *Money*
- *Other than money*
- *Both*

1. Have you ever paid for sex

- *No*
- *Yes, more than 6 months ago*
- *Yes, in the last 6 months*

1. Did you have sex with someone born in a foreign country in the last 6 months?

- *No (proceed to question 48)*
- *Yes*

1. where did this/these person(s) came from? (multiple answers possible)

- *Belgium*
- *Germany*
- *France*
- *United Kingdom*
- *Other*

1. You selected other. Where were this/these person(s) born? (in case you don’t know, please enter “unknown”

____________________

1. Did you ever used alcohol and/or drugs before or during sex?

- *No (proceed to question 51)*
- *Yes, longer than 6 months ago (proceed to question 51)*
- *Yes, in the past 6 months*

1. Which drugs did you use in the past 6 months before or during sex? (multiple answers possible)

- *Weed/hash*
- *GHB/GBL*
- *Ketamine*
- *4FMP*
- *Cocaine*
- *Heroine*
- *Speed*
- *XTC/MDMA*
- *Poppers*
- *Alcohol*
- *other drugs*

1. Which drugs other than just mentioned, did you taker in the past 6 months? (only if question 49 answered by “other drugs”)

____________________

1. Did you smoke (tabacco) in the past 6 months?

- *No*
- *Yes*

1. Are you allergic to antibiotics?

- *No (proceed to question 54)*
- *Yes*

1. Please specify the antibiotic (name of medicine) you are allergic for

____________________

1. Did you use antibiotics in the past month?

- *No*
- *Yes*

1. Please specify which antibiotic you used in the past month (only if question 54 answered by “yes”)

____________________

1. Are you taking medicines?

- *No*
- *Yes*

1. Please specify which medicines you take (only is question 56 answered by “yes”)

____________________

1. Are you vaccinated for hepatitis B?

- *No*
- *Yes*
- *I have had hepatitis B*
- *I don’t know*

1. Do you ever use an anal douche (enema)?

- *No*
- *Yes*

1. Did you ever experience sexual problems? (multiple answers possible)

- *No*
- *Yes, by pain*
- *Yes, by unwanted touching*
- *Yes, by less sexually aroused/no erection*
- *Yes, by early ejaculation*
- *Yes, by other*

1. Please specify shortly other sexual problems you experience (only is question 60 answered by “yes, by other”)

____________________

1. Are you planning to get tested again this year?

- *Definitely not*
- *Probably not*
- *Neutral*
- *Probably*
- *Definitely*

1. Did you experience one or more of the following symptoms in the past month? (symptoms on/around the genital area)

|  | *In the past month* | *At this moment* | *I dont have these symptoms* |
| --- | --- | --- | --- |
| *Pain/burning sensation during/after peeing* |  |  |  |
| *Peeing more often than usual* |  |  |  |
| *Blood in urine* |  |  |  |
| *Pain lower stomach during/after sexual contact* |  |  |  |
| *Pain in lower stomach not related to sexual contact* |  |  |  |
| *Blood loss during/after sexual contact* |  |  |  |
| *Discharge from penis* |  |  |  |
| *Genital itch* |  |  |  |
| *Wounds, blisters on the genitals* |  |  |  |
| *Warts on the genitals* |  |  |  |
| *Swollen genitals* |  |  |  |
| *Painful genital after/during sexual contact* |  |  |  |
| *Painful genitals not related to sexual contact* |  |  |  |

1. Did you experience one or more of the following symptoms in the past month? (symptoms on/around the anus)

|  | *In the past month* | *At this moment* | *I dont have these symptoms* |
| --- | --- | --- | --- |
| *Discharge* |  |  |  |
| *Bleeding during/after sexual contact* |  |  |  |
| *Bleeding not related to sexual contact* |  |  |  |
| *Wounds/blisters* |  |  |  |
| *Wrats* |  |  |  |
| *Swellings* |  |  |  |
| *Pain during/after sexual contact* |  |  |  |
| *Pain not related to sexual contact* |  |  |  |

1. Did you experience one of more of the following symptoms in the past month? (other symptoms)

|  | *In the past month* | *At this moment* | *I dont have these symptoms* |
| --- | --- | --- | --- |
| *Sore throat (not related to a cold)* |  |  |  |
| *Fever* |  |  |  |
| *Other symptoms* |  |  |  |

1. Please specify shortly which other symptoms you are now experiencing of experienced in the past month (only is question 65.3 not answered by “I don’t have these symptoms”)

____________________

1. Why do you want to test yourself? (multiple answers possible)

- *To prevent infection with partners*
- *For my own health*
- *I am experiencing symptoms*
- *For a periodic check*
- *I have been warned by someone*

1. Did you need additional explanation at the MUMC when you received the package?
   - *No, the information letter is clear*
   - *That would have been nice, but not necessary*
   - *Yes, that is necessary*
2. How clear were the instructions for the oral, urine, anal and blood test?

|  | *Easy to understand* | *Quite understandable* | *Difficult to understand* |
| --- | --- | --- | --- |
| *Oral* |  |  |  |
| *Urinal* |  |  |  |
| *Anal* |  |  |  |
| *Blood* |  |  |  |

1. What are for you important benefits of an STD test if you test this war (self-test)? (multiple answers possible)

- *That I can test myself when it suits me*
- *That I am able to test myself aat home*
- *That I can take a test without the chance of seeing someone*
- *That I don’t have to talk to a doctor*
- *I don’t need to travel to a specific location*
- *Other*

1. You selected other. What are important benefits for you?

____________________

1. If you would like to do an STD test again later, which way do you prefer?
   - *Visit to the GGD*
   - *Visit to the GP (general practitioner / huisarts)*
   - *Ordering a self-taking test (like this one) and sent it to the GGD*
   - *Ordering a commercial test on the internet / buying at drug store*
   - *Visit to the specialist (hospital)*
2. Would you give a test like this one to a friend so he or she can test themselves? (multiple answers possible)

- *No*
- *Yes, to my best friend*
- *Yes, to a good friend*
- *Yes, to a acquintance*
- *Yes, to a permanent (sex)partner*
- *Yes, to family*
- *Yes, to a fuck bddy*
- *Yes, to a one-night stand*
- *I don’t know to who I want to give it*

1. You selected no. Can you give us a reason? (multiple answers possible)

- *I am ashamed of that*
- *that’s private business for everybody*
- *I think they do not need it*
- *They won’t be able to test themselves properly*
- *I don’t know anyone to whom I can give the testkit*
- *Other*

1. You selected other. Can you give us an another reason why? (only is question 74 answered by “other”)

____________________

1. You selected yes. Why would you like to give a testkit to someone you know? (multiple answers possible) (only is question 73 not answered by “no”)

- *For protecting their health*
- *In case they are warned for an STD*
- *It will be useful when they experience symptoms*
- *If they want to test periodically*

1. The questions of the test kit have now been completed. The GGD regularly conducts research to improve our care. We often receive questions about drugs from visitors. We want to improve our knowledge about this and thus in the future we would like to invite you to fill in a questionnaire. Even if you do not have experience with drugs, your participation is valuable. Participation in this questionnaire is entirely without obligation and data will be treated confidentially. Can we send you this questionnaire.

- *Yes, I would like to participate in the future via mail*
- *Yes, I would like to participate in the future via SMS*
- *No, I would not like to participate in the future*
